# Building an integrated knowledge translation (IKT) evidence base: colloquium proceedings and research direction

**DOI:** 10.1186/s12961-019-0521-3

**Published:** 2020-01-20

**Authors:** L. Boland, A. Kothari, C. McCutcheon, I. D. Graham, for the Integrated Knowledge Translation Research Network

**Affiliations:** 1 School of Health Studies, Western University, 1151 Richmond Street, London, ON N6A 3K7 Canada; 2 Ottawa Hospital Research Institute, 501 Smyth Road, Ottawa, ON K1H 8L6 Canada; 3 School of Epidemiology and Public Health Faculty of Medicine, University of Ottawa, 307D-600 Peter Morand Cresent, Ottawa, ON K1G 5Z3 Canada

**Keywords:** Integrated knowledge translation, Knowledge users, Colloquium, Research co-production, Research agenda

## Abstract

**Background:**

Integrated knowledge translation (IKT) is a model of research co-production, whereby researchers partner with knowledge users throughout the research process and who can use the research recommendations in practice or policy. IKT approaches are used to improve the relevance and impact of research. As an emerging field, however, the evidence underpinning IKT is in active development. The Integrated Knowledge Translation Research Network represents a collaborative interdisciplinary team that aims to advance the state of IKT science.

**Methods:**

In 2017, the Integrated Knowledge Translation Research Network issued a call to its members for concept papers to further define IKT, outline an IKT research agenda, and inform the Integrated Knowledge Translation Research Network’s special meeting entitled, Integrated Knowledge Translation State of the Science Colloquium, in Ottawa, Canada (2018). At the colloquium, authors presented concept papers and discussed knowledge-gaps for a research agenda and implications for advancing the IKT field. We took detailed field notes, audio-recorded the meeting and analysed the data using qualitative content analysis.

**Results:**

Twenty-four participants attended the meeting, including researchers (*n* = 11), trainees (*n* = 6) and knowledge users (*n* = 7). Seven overarching categories emerged from these proceedings – IKT theory, IKT methods, IKT process, promoting partnership, definitions and distinctions of key IKT terms, capacity-building, and role of funders. Within these categories, priorities identified for future IKT research included: (1) improving clarity about research co-production/IKT theories and frameworks; (2) describing the process for engaging knowledge users; and (3) identifying research co-production/IKT outcomes and methods for evaluation.

**Conclusion:**

The Integrated Knowledge Translation State of the Science Colloquium initiated a research agenda to advance IKT science and practice. Next steps will focus on building a theoretical and evidence base for IKT.

## Background to the meeting

Integrated knowledge translation (IKT) is a model of research co-production whereby researchers partner with knowledge users who can use or implement the research recommendations and/or findings [1]. According to the Canadian Institutes of Health Research, IKT is a research approach that involves having knowledge users meaningfully partner on the research team [2]. Ideally, knowledge users are involved from study conception (e.g. defining the research question) to applying and disseminating the findings (e.g. publication). Theoretically, IKT represents a paradigm shift from solely scientist-driven research to collaborative problem-based research involving researchers and knowledge users generating real-life solutions to complex problems [3]. Involving knowledge users, or individuals who can use the research evidence to inform policy and practice decisions, requires partnership in the research process as well as significant involvement and influence from knowledge users [4]. Each stage in the research process is an opportunity for collaboration with knowledge users, including the development of the research questions, selection of the study design and methodology, ethics, tool development, selection of outcome measures, data collection, interpretation of the findings, crafting of the message for various audiences, dissemination and application. IKT is a recognised and accepted tenet of knowledge translation that purports to increase the relevance, applicability and impact of research results [5].

Involving knowledge users, or individuals who can use the research evidence to inform policy and practice decisions, requires meaningful partnership in the research process as well as significant involvement and influence from knowledge users [6]. Each stage in the research process is an opportunity for collaboration with knowledge users, including the development of the research questions, selection of the methodology, tool development, selection of outcome measures, data collection, interpretation of the findings, crafting of the message for various audiences, dissemination and application.

Although the research evidence underpinning IKT processes is promising, the field is in its infancy and several knowledge gaps remain. An evaluation of funding programmes to support knowledge translation showed that, compared to the traditional researcher-centred approach, co-produced research involving researchers and knowledge users was perceived to be more likely to improve the health of Canadians, create more effective health services and products, and strengthen the healthcare system [3]. A scoping review that evaluated knowledge user engagement in rehabilitation research found knowledge gaps in how knowledge users were identified and involved in the IKT process [7]. Another scoping review that examined IKT practices found that IKT activities were poorly reported and not theory based, and that the beneficial outcomes of IKT approaches remained unclear [8].

To address knowledge gaps and improve the state of IKT science, the Integrated Knowlege Translation Research Network (IKTRN) was founded in 2016 [6] (https://iktrn.ohri.ca/aboutus/who-we-are/). The purpose of this Network was to establish a collaborative interdisciplinary team to improve conceptual clarity, assess IKT impacts, develop theories and measurement tools, create capacity-building resources, and train researchers and knowledge users in IKT. The research network includes 42 IKT researchers, 29 knowledge users, 11 implementation scientists and 17 trainees with national and international linkages and collaborations. This group is well positioned to make significant contributions to IKT science.

## Methods

### Setting and participants

In 2017, the IKTRN issued a call to Network members to submit one-page proposals for producing concept papers of about 20–30 pages in length. The objective of the call was to further define the area of IKT, advance the understanding of IKT by focusing the IKTRN’s future research over the next few years, and inform the next IKTRN meeting. Proposals were reviewed by the executive committee (IDG, AK, CM) for alignment with the IKTRN’s mandate. The full papers were also peer reviewed. Authors who wrote a concept paper for the call (i.e., work that was not previously underway) received a $1500 CAD honorarium. Authors who already had papers underway were invited to have their work discussed with the other concept papers and be published in a forthcoming special issue.

In October 2018, the IKTRN hosted an invitational meeting in Ottawa, entitled IKTRN State of the Science Colloquium, of the lead authors of the concept papers and knowledge users. The purpose of the meeting was to share the ideas described in the concept papers, discuss their implications for advancing the field of IKT/research co-production and outline an IKTRN research agenda from the perspectives of researchers and knowledge users. Although research ethics board approval was not required for this report, all lead authors and knowledge users provided consent to publish their data, and reviewed and approved the final manuscript. Here, we report the proceedings of the 2018 IKT State of the Science Colloquium.

The format of the day-long meeting involved an opening keynote who reviewed the mandate of the IKTRN and activities to date. Then, attendees presented a 3-min electronic poster (*n* = 16) of their concept papers (see Additional file 1 for concept paper abstracts; Table 1) to stimulate discussion, which led to the overarching IKT categories and sub-categories. Subsequently, attendees were asked to break off into small groups to discuss the concept papers by categories (Table 1) and determine the research priorities. The small groups reassembled for a whole group research priority discussion.

**Table 1:** Characteristics of the concept papers presented at the meeting

Title	Objective	Findings/Relevance
IKT theory and ethics		
Providing clarity among approaches to partnered research: a multiphase mixed methods concept synthesis (AB 1.1)	To identify similarities and differences between IKT and other approaches to partnered research approaches	Several similarities and differences exist between IKT and other partnered research approaches Researchers should describe the purpose and processes for partnered research
Blending IKT and global health governance: a novel approach for wicked problems (AB 1.2)	To consider how combining IKT and global health governance could respond to wicked problems	IKT models should be expanded to include global health governance for addressing wicked problems
Why should we pay attention to power within research co-production approaches? (AB 1.3)	To define power as it relates to the co-production of health research, describe how it operates among and between various participant groups, and provide recommendations for achieving equitable partnerships	Attending to power imbalances at each stage of the research process is crucial in co-production; all participants should be aware of the implications of power imbalances and be supported to achieve and maintain a balance of power
Fostering the conduct of ethical and equitable research practices: the imperative for IKT in research conducted by and with Indigenous community members (AB 1.4)	To explore questions from Indigenous KUs’ perspectives on health research, researchers and research institutions	IKT shares commonality with and facilitates opportunities to further define and develop Indigenous knowledge translation; Western health systems need to expand and re-examine what constitutes ‘evidence’ in the development of useful and relevant knowledge
IKT methods		
Review protocol on IKT and partnerships: a coordinated multicenter team approach (AB 2.1)	To conduct five separate reviews to identify and synthesise the partnership literature and better understand the evidence base	These efforts will contribute to and improve the quality, conduct and reporting of research partnership literature
Conceptualizing IKT initiation: a meta-narrative review (AB 2.2)	To conduct a meta-narrative review of IKT initiation concepts, processes, enablers, barriers and outcomes in health services research, research from other disciplines and research traditions where IKT may be used	While IKT was recognised, it remains vaguely conceptualised despite lengthy research traditions; ongoing research of IKT initiation is needed to identify or generate relevant theory, and to establish outcomes and the determinants of those outcomes
Digital storytelling: a methodology for engaging vulnerable and marginalized populations in IKT (AB 2.3)	To present digital storytelling, an innovative approach to bring the voices of vulnerable and marginalised populations	Digital storytelling can uncover and amplify marginalised peoples’ voices for knowledge creation and translation
Community-based participatory research and integrated knowledge translation: advancing the co-creation of knowledge (AB 2.4)	To better understand the similarities and differences between community-based participatory research and IKT	When used appropriately, community-based participatory research and IKT both have the potential to contribute to, and advance, implementation science about the conduct of collaborative health systems research
IKT Evaluation and Impact		
Embracing complexity and uncertainty to create impact: exploring the emergence, processes and transformative potential of co-produced research (AB 3.1)	To identify a continuum of co-production processes and illustrate how it can influence interactions between research, policy and practice	This new ‘social model of impact’ and framework capture multi-layered and potentially transformative impacts of co-produced research
Advancing the evaluation of IKT (AB 3.2)	To explore how realistic evaluation could advance the field of IKT	A realist approach could contribute greatly to our ability to assess and increase the value of IKT
Exploring the synergies between focused ethnography and IKT (AB 3.3)	To describe our experience of conducting a focused ethnography with a collaborative IKT approach using a research exemplar about the experiences of frail, older adults undergoing a transcatheter aortic valve implantation	The integration of IKT with focused ethnography allows for real-time uptake of meaningful, emerging findings, the strengthening of collaborative research teams, and opportunities for sustained programmes of research and relationships in the field of health services research
Variable participation of KUs in cancer health services research: results of a multiple case study (AB 3.4)	To evaluate IKT activities within a cancer health services research network in Ontario, Canada	Barriers to KU co-production of cancer health services research include mismatched expectations of KU role and frequent KU turnover; research teams that take an IKT approach should consider specific strategies to address barriers to KU engagement
Translating research into action: an international study of the role of research funders (AB 3.5)	To provide an international review of health research funders’ efforts to encourage knowledge translation	Knowledge translation approaches and mechanisms vary across region and funder type; evaluation of funding agency efforts to promote and/or support knowledge translation should be prioritised and actioned
Patient engagement and IKT		
Patient engagement in IKT research (AB 4.1)	To explore how existing health research methodologies can inform current approaches to patient engagement and support meaningful engagement with patients in IKT research	This iterative and exploratory process will provide greater conceptual clarity and density around patient engagement within IKT research and make explicit how the principles, practices and outcomes of patient engagement and IKT overlap across the research process
Patient engagement and IKT research: what can be learned from qualitative health research methods? (AB 4.2)	To conduct a scoping review to explore how patients are engaged in IKT research and explore how IKT approaches can foster meaningful patient engagement	IKT researchers will increasingly need to engage patients as KUs to satisfy health policy, care and research mandates for engaging patients as partners in decision-making
Engaging frail and seriously ill patients in IKT (AB 4.3)	To systematically review and synthesise how those with serious illness and frailty have been involved as research partners	Given the fluctuations in health for frail and seriously ill patients, researchers need to be flexible and creative in their approach to patient engagement. Further research is required to build the evidence base about what it means to engage frail and seriously ill patients in research so that developing research is more applicable to their needs

*AB* abstract, *IKT* integrated knowledge translation, *KU* knowledge user

### Data collection and analysis

We categorised concept papers under the following headings to reflect their content and facilitate presentation flow: IKT theory and ethics, IKT methods, patient engagement and IKT, and IKT evaluation and impact. After the presentations, attendees broke into small groups to discuss the implications of the presentations for the field of IKT. Attendees then reassembled to present the main points of their small group discussions and prioritise the areas to guide an IKT research agenda. All presentations and discussions were documented using field notes and audio recordings.

We used qualitative content analysis to characterise the IKT overarching concepts that emerged from the discussions as well as priority directions for future IKT research [9]. First, a reviewer (LB) filled field note gaps by listening to the recordings of the entire proceeding. LB then read the field notes and in their entirety to gain a holistic sense of the data. During the second reading, LB segmented the text to reflect important information related to IKT and the objectives of the meeting (i.e. IKT concepts, implications and future research) and transferred this data into an excel spreadsheet. Subsequently, LB proposed a set of codes, finalised by consensus with IDG, and coded the data, which was then categorised based on recurring codes reflecting underlying patterns or indicating important issues for the participants. We checked our findings by sending the results to meeting participants to ensure our report accurately reflected participants’ discussions [10].

## Results

Twenty-four participants attended the meeting, including researchers (*n* = 11), knowledge users (*n* = 7) and trainees (*n* = 6) (see Additional file 2 for attendees’ biographies). Researchers and knowledge users mostly represented Canadian universities, funding agencies and provincial health services. Trainees include individuals at the masters, doctoral and postdoctoral level working with a supervisor or co-supervisor who is a member of the IKTRN and is engaged in research that uses an IKT approach or is contributing to the science of IKT. Member checking resulted in few minor edits (e.g. rephrasing) and all members agreed that the data was accurately represented.

### Research agenda

Seven overarching categories emerged from these proceedings: theory, methods, process, promoting partnership, definitions and distinctions, capacity-building, and role of funders (Table 2). Participants expressed that greater theoretical clarity was required, including guiding frameworks for using and evaluating IKT processes. Discussions revealed that deeper development of IKT methodological concepts, including designs, outcomes, evaluation tools and reporting standards would advance the IKT field. Additional guidance for both researchers and knowledge users about how to initiate and optimise the IKT process is needed. Similarly, strategies for promoting meaningful and equal partnerships between researchers and knowledge users, including addressing leadership and power imbalances, was identified as a priority. IKTRN members identified the need to define and create a common language for IKT as well as to distinguish it from other similar concepts (e.g. participatory research and patient/community engagement). The process of capacity-building for the IKTRN members, trainees and others was considered. Specifically, building IKT capacity among current IKT experts by developing a centralised repository to share resources and for developing the next generation of IKT experts (both researchers and knowledge users). Finally, exploring the role of funders in advancing IKT was important to participants.

**Table 2:** IKT categories derived from group discussions

Category	Sub-category	Description
IKT theory	Frameworks	IKT frameworks need to be identified, developed and validated
	Clarity	Greater theoretical clarity about IKT is required; the underlying assumptions of IKT need to be made explicit
IKT methods	Search filters	Search filters that identify IKT concepts in the literature are required
	IKT data collection	Development of approaches to obtaining meaningful data that illustrate the impact of IKT is required; considerations include embedded research from the health system and sampling from multiple populations for IKT outcomes
	Outcomes	Specific IKT outcomes need to be identified
	Evaluation	Tools for evaluating IKT need to be identified or developed and validated
	Reporting guidelines	Report standards for IKT research studies are required
IKT processes	Initiating IKT	Identify strategies to initiate engagement with KUs
	Describing KU	Describe who the KUs are that are using IKT processes
	Accountability	Identify who is accountable for the IKT process
	Indicators of success	Identifying indicators of IKT success
	Governance and leadership	Identify the governance structure for research required to support IKT
Promoting partnership	Level of partnership	Ensuring that KUs are involved in the research priority-setting and engaged with at the highest level of partnership; ensuring engagement is genuine and not tokenism
	Diversity	Accounting for diverse perspectives in the partnership process of IKT
	Experiences	Need for detailed reporting on the characteristics and experiences of various knowledge user types involved in the IKT partnership
	KU needs	Identifying and supporting the KU’s needs throughout the partnership
	Safe spaces	Ensure safe participatory spaces for KUs
	Researcher reciprocation	Identifying ways for researchers to meet the needs of vulnerable groups
	KU reimbursement and IKT cost	Reimbursing KUs for their time and costs associated with collaboration
Definitions and distinctions	Distinguishing IKT from other concepts and approaches	Define and distinguish IKT from patient-oriented research, quality improvement, other designs and methods, learning health systems, implementation science
Capacity-building	Repository	Create a repository of IKT frameworks, tools and publications
	Trainees	Develop a vision for building IKT capacity in trainees
Role of funders	Funding and costs	Examining how funders should account for the added cost of IKT
	Funders as KUs	Determining how and whether funders should be KUs
	Peer-review	Exploring the impact of peer-reviewers on grant success
	Structures	Changing the funding structures to support IKT and KU partnership

*IKT* integrated knowledge translation, *KU* knowledge user

## Discussion

Discussions lead to the emergence of a preliminary agenda for future research primarily aimed within the IKTRN, but also informative for other IKT scholars and knowledge users interested in advancing the field. Main research agenda priorities within the overarching categories included (1) improving clarity about IKT theories and frameworks; (2) describing the process for engaging knowledge users in the IKT process; and (3) identifying IKT outcomes and methods for evaluation.

Attendees identified the need to develop a common understanding of theories and frameworks and how these are unique from, and overlap with, other collaborative or co-production approaches. IKT shares common principles with many collaborative research approaches, including co-production of knowledge, participatory research, linkage and exchange, knowledge production, engaged scholarship, patient engagement or involvement, and community-based research [11–17]. Given that terms could be used interchangeably despite subtle and potentially important differences suggests a need to make explicit how IKT concepts and processes are similar to, and unique from, existing collaborative research approaches. A common understanding of IKT’s terms, theories and frameworks will provide researchers and knowledge users common concepts and vocabulary, the ability to better synthesise and compare the literature, and a more systematic understanding of IKT. Work is required to develop, test and refine IKT theories and frameworks.

The research agenda should also better describe the role, experiences and processes for partnering with knowledge users in IKT. As such, the contextual factors that hinder or enable knowledge user partnership in IKT processes, and the influence of leadership and power relations on knowledge users’ ability to meaningfully contribute to the research process or mitigating power imbalances, are key areas for investigation. Such research should lead to recommendations to guide researchers and knowledge users in the optimal use of IKT approaches.

Another priority for the research agenda is to evaluate the impacts of IKT. This involves determining appropriate IKT outcomes that reflect a meaningful collaborative approach, identifying or developing valid and reliable instruments to measure IKT processes and outcomes, and establishing reporting standards for IKT.

## Conclusions

By partnering with knowledge users in the research process, the young field of IKT has potential to improve the relevance, impact and efficiency of research. The presentation of concept papers and subsequent discussion initiated a research agenda to advance IKT science and practice, both within the Network and for the broader community. Next steps for the IKTRN will include mobilising network members and other researchers around the identified research agenda.

## Supplementary information

**Additional file 1.** List and bio-sketch of IKT meeting attendees.

**Additional file 2.** Concept paper abstracts presented at the IKTRN meeting.

## Data Availability

Not applicable.

## Abbreviations

IKT: Integrated knowledge translation
IKTRN: Integrated Knowledge Translation Research Network
