# Supplementary material for: Building an integrated knowledge translation (IKT) evidence base: colloquium proceedings and research direction
Source: Health Res Policy Syst. 2020 Jan 20;18:8. doi: 10.1186/s12961-019-0521-3 (PMC6972018; doi:10.1186/s12961-019-0521-3)
Supplement: Supplementary file 1 — Additional file 1. List and bio-sketch of IKT meeting attendees. [file 12961_2019_521_MOESM1_ESM.docx]

**Additional file 1.** List and bio-sketch of IKT meeting attendees

**Laura Boland**

Laura Boland is a speech-language pathologist and is a Post-Doctoral Fellow with the Integrated Knowledge Translation Network at the Ottawa Hospital Research Institute (OHRI) and Western University. Her research interests include using integrated knowledge translation approaches to study the implementation of shared decision making in pediatric clinical practice. Laura was involved in the development, evaluation, and implementation of multiple shared decision making interventions at the Children’s Hospital of Eastern Ontario and has provided shared decision making training to over 200 healthcare providers. Her post-doctoral fellowship is focused on developing and evaluating a pediatric shared decision making implementation program for healthcare providers, parents, and children. Laura is also a member of the Patient Decision Aid Research Group and the Center for Implementation Research at the OHRI, as well as the International Patient Decision Aids Standards Collaboration (IPDAS).

**Christine Cassidy**

Christine Cassidy, RN, PhD, is a registered nurse and CIHR Health System Impact Postdoctoral Fellow at the IWK Health Centre and University of Ottawa. She completed her BScN at the University of Prince Edward Island and PhD in Nursing at Dalhousie University where she studied the development of behaviour change interventions. Her doctoral work was funded by a CIHR Frederick Banting and Charles Best Doctoral Research Award and Killam Predoctoral Scholarship. She is also the recipient of the College of Registered Nurses of Nova Scotia Rising Star Award. Dr. Cassidy’s current research focuses on using a theory-based approach to implement and evaluate nursing practice changes in pediatric care. For her postdoctoral fellowship, she is using integrated knowledge translation to implement a pediatric early warning system at the IWK Health Centre and examine the role and process of facilitation during implementation.

**Wendy Gifford**

Wendy Gifford (RN, PhD) is Associate Professor at University of Ottawa School of Nursing and Co-Director of Center for Research on Health and Nursing. Her research focuses on leadership for knowledge translation with health care providers to improve healthcare delivery and patient outcomes. She is working with Indigenous Peoples to support culturally safe cancer survivorship care, and with nurses and care aids in long-term care homes to implement falls prevention. International KT collaborations include Sweden and China. Dr. Gifford’s clinical career included outpost nursing in First Nations communities in Yukon Territory.

**Ian Graham**

Dr. Ian Graham is Senior Scientist in the Clinical Epidemiology Program of the Ottawa Hospital Research Institute and Professor in the department of Epidemiology & Community Medicine, of the University of Ottawa. From 2006-2012 he was on an interchange with the Canadian Institutes of Health Research where he held the position of Vice-President of the Knowledge Translation and Public Outreach. Dr. Graham obtained a Ph.D. in medical sociology from McGill University. His research focuses on knowledge translation and conducting applied research on strategies to increase implementation of evidence-informed practice. He has published over 250 peer reviewed articles and is co-editor of Knowledge Translation in Health Care (2009) and Evaluating the Impact of Implementing Evidence-based Practice (2010). Ian was awarded the Queen Elizabeth II Diamond Jubilee Medal for his “leadership contributions to CIHR and for changing the way research knowledge is used and demonstrating to funding agencies around the world how to move knowledge into action.” In 2015, Dr. Graham was awarded a seven-year CIHR Foundation Grant that became the Integrated Knowledge Translation Research Network.

**Karen Harlos**

Dr. Karen Harlos is Professor and past Inaugural Chair, Department of Business, University of Winnipeg. Her field is organizational behaviour and she researches workplace bullying and mistreatment, how employees respond, and worklife quality. Dr. Harlos has led and served on several large research teams. Currently, she leads a 4-year project on workplace bullying and mistreatment prevention funded by SSHRC in four large Manitoba organizations with Canadian and international scholars ([www.uwinnipeg.ca/wbmp](http://www.uwinnipeg.ca/wbmp)). Her published work appears in numerous academic journals and she has presented at conferences and workshops around the world. She also trains and consults on conflict resolution, teamwork, leadership, and psychologically healthy workplaces ([www.karenharlos.com](http://www.karenharlos.com))

**Janet Jull**

Dr. Janet Jull is an Assistant Professor in the Doctor of Science in Rehabilitation and Health Leadership Program at the Queen's University School of Rehabilitation Therapy (Kingston, Ontario) and Affiliate Investigator at the Ottawa Hospital Research Institute (Ottawa, Ontario). Janet develops and evaluates shared decision making tools and approaches to support client-centred care in partnerships with First Nations, Inuit and Métis communities, with a particular focus on cancer care. Janet also investigates how research is conducted in partnerships; specifically when those who are engaged in in the production of research partner with those who contend with the real-world needs and constraints of health systems and their users.

**Anita Kothari**

Anita Kothari is an Associate Professor in the School of Health Studies at the University of Western Ontario. She has two main areas of research expertise. First, her work focuses on developing a greater understanding of knowledge translation (KT) in community-based organizations and identifying the factors that influence the successful implementation of KT initiatives into practice. She involves multiple community members, researchers, and government agencies at all stages of the research process. That is, she facilitates partnerships and develops capacity in ways that will result in research findings that can be applied. Kothari’s second area of research expertise is in public health systems research. Specifically, she addresses the needs of socially excluded and marginalized populations by studying how public health programs are organized and delivered in communities.

**Sara Kreindler**

Sara Kreindler is Manitoba Research Chair in Health System Innovation and Assistant Professor with the University of Manitoba's Department of Community Health Sciences, and a member of the George & Fay Yee Centre for Healthcare Innovation. Her multi-method research centres on the question of how best to design and implement models of health-services delivery, integrating technical and social considerations within a system perspective. She is currently leading two decision-maker–partnered research projects: a Western Canadian study to understand the drivers of inter-regional variation in patient flow performance, and a provincial study of primary care renewal. She is also working on a conceptual model to explain the outcomes of attempted system change. A Rhodes Scholar, Sara completed her doctorate in Social Psychology at Oxford University in 2004. Prior to her faculty appointment, she spent eight years as an embedded researcher with the Winnipeg Regional Health Authority, and was a Harkness Fellow.

**Susan Law**

Dr. Susan Law is Director of Research and Scientist at the Trillium Institute for Better Health, Mississauga, and Associate Professor at IHPME, University of Toronto. She leads the Canadian Health Experiences initiative as part of an international collaboration that focuses on personal narratives of health and illness, using qualitative research and video recording (see: www.healthexperiences.ca; www.dipexinternational.org). Susan is also involved in research on Indigenous and global health, health services research, knowledge translation, and patient engagement. She has an MHSc in health administration (University of Toronto) and a PhD from LSHTM (University of London UK).

**Claire Ludwig**

Claire Ludwig is a PhD candidate at the School of Nursing, Faculty of Health Sciences, University of Ottawa. Her doctoral work is focused on patient engagement in research, specifically engaging frail and/or seriously ill patients as knowledge users. Her other research interests examine how patients and nurses negotiate the process of triage and self-management in cancer symptom management. Claire is a senior health care leader involved in the development, implementation and evaluation of large-scale programs aimed at improving patient and caregiver outcomes. Claire is also a patient with cancer currently receiving immunotherapy treatment. She has served as a knowledge user on research projects as a health care administrator and as a patient advisor in the acute treatment phase of her illness.

**Robert McLean**

Robert McLean, M.Sc., B.A., B.Com., is currently Senior Program Specialist in Evaluation at Canada’s International Development Research Centre (IDRC). Rob has over 10 years of professional evaluation experience, the majority related to research quality, translation, and impact evaluation. He is co-author of the Research Quality Plus (RQ+) approach, CIHR’s Evaluation of KT funding, and currently leading a research project on Scaling Research Impact. He was the Chair of the Evaluation Committee of the Global Alliance for Chronic Diseases. He has published in health sciences and evaluation books and journals, and has been an invited speaker on research evaluation internationally including for the United Nations. Rob is an active member of the Canadian, American, and South Asian Evaluation Associations. He is PhD candidate in the Faculty of Medicine and Health Sciences at Stellenbosch University, South Africa. His PhD is supervised by Dr Ian Graham and Dr Jimmy Volmink.

**Chris McCutcheon**

Chris McCutcheon is a knowledge translation expert who specializes in IKT and health services and policy research. He is currently the manager of the Integrated Knowledge Translation Research Network at the Ottawa Hospital Research Institute. For close to a decade he designed and managed applied research programs for the Canadian Health Services Research Foundation and authored several of their dissemination products. From 2007 to 2010 Chris worked for the Knowledge Translation portfolio of the Canadian Institutes of Health Research (CIHR). While at CIHR, Chris managed IKT research programs, such as Partnerships for Health System Improvement and the Knowledge Synthesis grants. He also designed and piloted Evidence on Tap, CIHR’s first research program designed to produce rapid and relevant research for health-system decision makers. Chris holds a master’s degree in Social and Political Thought from York University.

**Martha Mcleod**

Martha MacLeod, PhD, RN, is Professor at the University of Northern British Columbia where she is the Northern Health – UNBC Knowledge Mobilization Research Chair and Co-Leads UNBC’s Health Research Institute. Martha takes a qualitative, partnered approach to exploring how knowledge is created and taken up in health services, particularly in sparsely populated areas. She has led two national studies on rural and remote nursing and is currently examining how primary healthcare transformation occurs in a rural and northern health authority, including how middle and front line managers actually lead organizational changes. Notably, she is leading a new study on what it means to take a hermeneutic approach to implementation science. Martha has published and presented widely on rural and northern practice, education, leadership, knowledge translation, and network development. Martha regularly engages with researchers, practitioners, health service leaders, and policy-makers in advancing regional, national, and international research and knowledge translation networks.

**Melody Morton-Ninomiya**

Melody Morton-Ninomiya is a Project Scientist at the Institute for Mental Health Policy Research, Centre for Addiction and Mental Health (CAMH). She currently co-leads a number of community-driven mental health and wellness projects in First Nation communities across Ontario and Labrador. Her background in education, restorative justice, and community health inform her research in the area of Indigenous health and well-being, knowledge translation, and Indigenous health research principles and practices. Prior to working at CAMH, Melody worked at Well Living House, an action research centre focused on Indigenous health and well-being at St. Michael’s Hospital in Toronto, in the area of Indigenous knowledge translation.

**Kelly Mrklas**

Kelly Mrklas is a KT Implementation Scientist, Strategic Clinical Networks™ in the System Innovation & Programs portfolio at Alberta Health Services. Kelly helps clinicians, researchers, decision-/policy makers, health care leaders and staff turn new evidence, innovations and improvements into daily work. Kelly designs and troubleshoots implementation strategies to optimize their efficiency, effectiveness and sustainability, in diverse contexts. Since 2014, Kelly has conducted more than 260 consults, 80 presentations, and collaborated on over 60 grant submissions. She is currently a named investigator, collaborator, and/or knowledge user on 24 major research initiatives totaling over $22M and leads/co-leads several research initiatives in knowledge synthesis, integrated KT, implementation, and sustainability. Kelly developed the first campus-wide KT consultation service at the University of Calgary in 2010. Her dissertation focuses on expanding the evidence base and identifying tools for health research partnership outcomes and impact assessment.

**Tram Nguyen**

Tram Nguyen, PhD, is a postdoctoral fellow working with Dr. Ian Graham in the School of Epidemiology and Public Health at the University of Ottawa. She is a member of the Integrated Knowledge Translation Network and collaborator with the Ottawa Hospital Research Institute. She is also affiliated with the CanChild Centre for Childhood Disability Research as well as the School of Rehabilitation Science at McMaster University. Her main areas of expertise are in integrated knowledge translation, pediatric rehabilitation, healthcare transitions for youth with disabilities and chronic health conditions, and professional language development. The objectives of her postdoctoral research include: i) integrated knowledge translation as well as other approaches to collaborative research, ii) perspectives of youth with disabilities and researchers about experiences, satisfaction, contribution, and impact of research partnerships, and iii) identifying key elements of the partnering process to contribute to the development of a partnership evaluation tool.

**Mary Ann O’Brien**

Mary Ann O’Brien is an Assistant Professor in the Department of Family and Community Medicine, University of Toronto, and Scientific Associate with the Knowledge Translation Research Network, Health Services Research Network, Ontario Institute for Cancer Research. Her research interests focus on knowledge translation related to cancer. Specific interests include: 1) patient-physician communication and decision making in breast cancer, 2) prevention of cancer and other chronic diseases in primary care, and 3) the effectiveness of interventions to improve health professional practice including quality improvement strategies focused on care for patients with breast cancer. She is an Associate Editor with the Cochrane Effective Practice and Organisation of Care Review Group, Knowledge Translation Section Editor for the journal Current Oncology, and Digest Editor, Canadian Association for Psychosocial Oncology.

**Katrina Plamondon**

Katrina Plamondon is an RN and Practice Leader for Research and Knowledge Translation at Interior Health (British Columbia), where her work focuses enabling people across the system to use and do research. Her clinical foundations are in critical care/emergency and street outreach. Katrina has a Master of Science in Community Health & Epidemiology (University of Saskatchewan) and is in the final stages of a PhD at the University of British Columbia under a Banting & Best Canada Graduate Scholarship. Her doctoral work extends a decade of research and practice in knowledge translation for health equity. A long-time member of the Canadian Coalition for Global Health Research, Katrina co-chairs the Policy & Advocacy Committee to promote more consistent, coherent investments and practices in global health research. She was the Principal Investigator for a multi-year study that led to the creation of equity-centred CCGHR Principles for Global Health Research (available here: [www.ccghr.ca/resources/principles-global-health-research/](http://www.ccghr.ca/resources/principles-global-health-research/)).

**Gayle Scarrow**

Gayle Scarrow is the Director, Knowledge Translation at the Michael Smith Foundation for Health Research (MSFHR). She leads the development, implementation, evaluation and ongoing management of MSFHR's knowledge translation plan for the purpose of fostering and accelerating the impact of health research in BC and beyond. She has held numerous roles in health care and health research for the past 30 years including as a radiation technologist, research coordinator, research writer, KT manager and, through her work with MSFHR, as a knowledge user on various research grants to both contribute to the academic KT literature and to inform MSFHR’s KT work.

**Kathryn Sibley**

Dr. Kathryn Sibley, or Kate as she is known, holds a Canada Research Chair in Integrated Knowledge Translation in Rehabilitation Sciences at the University of Manitoba. She an Assistant Professor in the Department of Community Health Sciences, Max Rady College of Medicine, and cross-appointed in the College of Rehabilitation Sciences. She is also the Director of the Knowledge Translation platform at the Centre for Healthcare Innovation, a partnership between the U of M and Winnipeg Regional Health Authority. The goals of her research program are to (i) increase use of evidence-based rehabilitation strategies in real-world settings, (ii) advance implementation science through a rehabilitation context, and (iii) advance the science and practice of integrated knowledge translation. Kate holds a BSc in Kinesiology from the University of Waterloo and graduate degrees in Rehabilitation and Medical Sciences from the University of Toronto. She also completed postdoctoral training in Knowledge Translation at the Toronto Rehabilitation Institute.

**Sarah Wu**

Sarah Wu is a PhD Candidate at the University of Waterloo and an Alzheimer Society of Canada Doctoral Fellow. Sarah’s doctoral training includes working collaboratively with those who live and work in long-term care homes in the development and implementation of relationship-centred interventions to improve the mealtime experience. Her own thesis research looks to explore how meals in this setting can become more relational to include family members in mealtime processes. Sarah sits on the Board of Directors for the Toronto Council on Aging and is a subject matter expert for the Ontario Retirement Communities Association’s Dementia Task Force.

**Lesley Wye**

Lesley's current research interests include: knowledge exchange, commissioning and end of life care. Other research interests include: community services, quality initiatives (e.g. CQUIN), and complementary therapies. Her methodological interests are qualitative and mixed methods studies in service evaluations and realistic evaluations. She is currently leading on a NIHR funded study of knowledge exchange between clinical commissioners and external providers (private and not-for-profit) due to finish in February 2014. She is a co-applicant on a NIHR National School for Primary Care Research study investigating general practice factors that contribute to unscheduled hospital admissions. She co-supervises a PhD student looking at universal consent in primary care research. Her teaching responsibilities include tutoring on the first year Society, Health and Medicine course and running electives on acupuncture and other complementary therapies. She also co-edits the website for the Centre for Lesley leads the [Knowledge Mobilisation](http://www.bristol.ac.uk/primaryhealthcare/km/who-we-are/) team working across the School for Social and Community Medicine and local commissioning organisations.

**Maria Zych**

Maria Zych is a part-time MSc student at the Institute of Health Policy, Management and Evaluation, University of Toronto (UofT). Her interests include organizational management of resources for better knowledge translation in health care organizations, and multidisciplinary roles in integrated knowledge translation (IKT) initiatives. She currently works as a librarian at the U of T Faculty of Dentistry, where she offers instructional workshops including information retrieval using health sciences databases, citation management systems, finding information at the point of care, and other tasks. The increased demand for literature searching to create syntheses such as systematic reviews and clinical practice guidelines that can guide knowledge translation inspired her to pursue graduate studies and expand her knowledge of knowledge translation.
