# Supplementary material for: Building an integrated knowledge translation (IKT) evidence base: colloquium proceedings and research direction
Source: Health Res Policy Syst. 2020 Jan 20;18:8. doi: 10.1186/s12961-019-0521-3 (PMC6972018; doi:10.1186/s12961-019-0521-3)
Supplement: Supplementary file 2 — Additional file 2. Concept paper abstracts presented at the IKTRN meeting. [file 12961_2019_521_MOESM2_ESM.docx]

**Additional file 2.** Concept paper abstracts presented at the IKTRN meeting

***Keynote: IKTRN Protocol***

**Moving knowledge into action for more effective practice, programmes and policy: protocol for a research programme on integrated knowledge translation**

Graham I, Kothari A, McCutcheon C *et al.*

**Background**

Health research is conducted with the expectation that it advances knowledge and eventually translates into improved health systems and population health. However, research findings are often caught in the know-do gap: they are not acted upon in a timely way or not applied at all. Integrated knowledge translation (IKT) is advanced as a way to increase the relevance, applicability and impact of research. With IKT, knowledge users work with researchers throughout the research process, starting with identification of the research question. Knowledge users represent those who would be able to use research results to inform their decisions (e.g. clinicians, managers, policy makers, patients/families and others). Stakeholders are increasingly interested in the idea that IKT generates greater and faster societal impact. Stakeholders are all those who are interested in the use of research results but may not necessarily use them for their own decision-making (e.g. governments, funders, researchers, health system managers and policy makers, patients and clinicians). Although IKT is broadly accepted, the actual research supporting it is limited and there is uncertainty about how best to conduct and support IKT. This paper presents a protocol for a programme of research testing the assumption that engaging the users of research in phases of its production leads to (a) greater appreciation of and capacity to use research; (b) the production of more relevant, useful and applicable research that results in greater impact; and (c) conditions under which it is more likely that research results will influence policy, managerial and clinical decision-making.

**Methods**

The research programme will adopt an interdisciplinary, international, cross-sector approach, using multiple and mixed methods to reflect the complex and social nature of research partnerships. We will use ongoing and future natural IKT experiments as multiple cases to study IKT in depth, and we will take advantage of the team’s existing relationships with provincial, national and international organizations. Case studies will be retrospective and prospective, and the 7-year grant period will enable longitudinal studies. The initiation of partnerships, funding processes, the research lifecycle and then outcomes/impacts post project will be studied in real time. These living laboratories will also allow testing of strategies to improve the efficiency and effectiveness of the IKT approach.

**Discussion**

This is the first interdisciplinary, systematic and programmatic research study on IKT. The research will provide scientific evidence on how to reliably and validly measure collaborative research partnerships and their impacts. The proposed research will build the science base for IKT, assess its relationship with research use and identify best practices and appropriate conditions for conducting IKT to achieve the greatest impact. It will also train and mentor the next generation of IKT researchers.

***Theme 1: IKT Theory and Ethics***

**1.1 Providing clarity among approaches to partnered research: A multiphase mix methods concept synthesis**

Nguyen T, Graham ID, Bowen S, Cargo M, Estabrooks C, Grimshaw J, Kothari A, Lavis J, Macaulay A, MacLeod M, Mrklas K, Phipps D, Ramsden V, Renfrew M, Salsberg J, Straus S

**Background**

It is of paramount importance to distinguish differences and similarities among current approaches to partnered research to provide clarity by highlighting the unique features of each approach.

**Purpose**

To identify similarities and differences between integrated knowledge translation and other approaches to partnered research: co-production/co-creation, participatory research, engaged scholarship, and modes of knowledge production.

**Methods**

A modified concept synthesis using a multiphase mix methods iterative approach. In the first phase, a cursory literature review was conducted to identify current approaches to partnered research and germinal publications (peer-reviewed published and grey literature) related to each approach. An excel table was constructed to extract the following information from the findings of the review on each approach: definitions, historical roots, defining characteristics, and germinal publications. In the second phase, key informants/experts in each tradition were identified through germinal publications, networking and snowball sampling. Qualitative semi-structured interviews were then conducted with the15 key informants to gain insight into their perspectives and to suggest key papers in that tradition. Participants’ responses and suggested germinal publications allowed for additional insights into each approach which were synthesized to revise, modify, and validate the extracted data in the first phase. In the last phase, data from the first and second phases were analyzed together to develop a master table highlighting the differences and similarities among the approaches. Data triangulation and member-checking was conducted among the experts to ensure accuracy of the master table.

**Results**

The findings are among the first to provide clarity among approaches to partnered research. Although there are more similarities compared to differences, researchers should always describe the purpose and processes for partnered research.

**Conclusions**

The findings are instrumental in contributing to foundational knowledge about approaches to partnered research. These findings should be considered by all those interested in partnered research in health and beyond.

**1.2 Blending Integrated Knowledge Translation and Global Health Governance: A novel approach for wicked problems**

Plamondon K, Pemberton J

**Background**

Problems designated as ‘wicked’ earn their label from their inherent resistance to resolve. They are messy, dynamic problems that pose challenges for connecting knowledge to action. The persistence of health inequities is a wicked problem for which there is strong evidence of causal roots in the maldistribution of power, resources, and money within and between countries. Though the evidence is clear, the solutions are far from straightforward. Integrated knowledge translation (iKT) ought to be well suited for designing evidence-informed solutions; but current frameworks are limited in their capacity to navigate complexity.

**Discussion**

Connecting knowledge to action on root causes of health inequities implicates the structures and systems that shape how society is organized. This infinitely complex work demands sophisticated examination of drivers and disrupters of inequities and a vast imagination for who (and what) should be engaged. Current iKT models’ guidance for processes of co-creating and applying knowledge are effective in well-defined contexts, but utility becomes limited when applied to the amorphous political, social, and cultural environments of wicked problems. Specifically, these models lack consideration of issues of power and provide little direction for how to support cohesive efforts toward a common goal. Governance, however, may provide insight into these issues. Governance, particularly global health governance (GHG), is a polycentric system that provides a mechanism for collective problem-solving for improved health, through the interplay of different institutional forms and actors at different levels in pursuit of common goals. Contemplating the inclusion of governance in iKT requires re-examining roles, responsibilities, power, and voice in processes of connecting knowledge with action. We argue for expanding iKT models to include GHG as a means of considering the complexity of issues and open new possibilities for evidence-informed action on wicked problems.

**Conclusion**

Combining GHG and iKT could be an important driver of possibility for responding to wicked problems like health inequities. Attention to governance in iKT science, theory, and models is essential for advancing this evolving field. Integrated learning between these two fields, adopting principles of GHG alongside the strategies of iKT, is a promising opportunity to strengthen capacity to redress wicked problems.

- 1. **Why should we pay attention to power within research co-production approaches?**

Holmes B, Laing I, Kothari A, McCutcheon C, Graham I

**Background**

The co-production of research – where researchers partner with knowledge users through the research process — is gaining increasing attention in health-related work. This is a decades-old practice with roots in community-based participatory-action research (CBPAR) approaches and more recently known by other names including engaged scholarship, co-production of knowledge and integrated knowledge translation. The literature supporting the improvement of co-production practices is growing but little attention has so far been paid to potential power imbalances among co-production participants.

**Main body**

Since co-production is a partnership, power relationships are critical for all participants and stakeholders. Drawing on theory and practice, the purpose of this discussion paper is to further define power as related to the co-production of health research; describe how it operates among and between various participant groups (laypersons, researchers, practitioners, administrators); and provide recommendations for achieving equitable partnerships. Through examining scenarios, we identify unique issues related to formal and informal power in a range of co-production approaches that must be addressed for these research partnerships to achieve their potential through just means. We suggest that existing strategies aimed at creating true co-production do not fully acknowledge the power structures that exist within and beyond health research. Finally, we advocate for the study of co-production in action to help improve this important, growing area.

**Conclusion**

Attending to power imbalances at each stage of the research process is crucial in co-production. All participants should be aware of the implications of power imbalances and be supported to achieve and maintain a balance of power.

**1.4 Fostering the conduct of ethical and equitable research practices: the imperative for integrated knowledge translation in research conducted by and with Indigenous community members**

Jull J, Compton I, Morton-Ninomiya M, Picard A

**Background**

Indigenous people are affected by major health issues at much higher rates than for general populations, and Western health care models do not necessarily align with Indigenous values, knowledge systems, and care practices. Knowledge translation (KT) describes ways of moving knowledge from theory into health systems’ applications, although there are limitations and concerns related to how KT is practiced within Western research methods for Indigenous health contexts. Integrated KT, an approach to research that engages knowledge users with researchers throughout the entire research process, is congruent with Indigenous health research principles. In this paper, we raise and discuss questions from Indigenous knowledge-users’ perspectives on health research, researchers, and research institutions.

**Main**

We describe: 1) how integrated KT has been found to accommodate both Indigenous and Western-informed perspectives as well as community-research partnerships that support knowledge user collaboration throughout the research process; 2) how to define and uphold ethical practices that are agreed upon by those involved in a program of research; and 3) why it is necessary to co-create knowledge that gives voice to Indigenous community members within academic spaces. We argue that integrated KT, as a collaborative research practice, can create opportunities and space for different knowledges to coexist and improve health systems.

**Conclusion**

The use of integrated KT shares commonality with and facilitates opportunities to further define and develop Indigenous KT. Western health systems need to expand and re-examine what constitutes “evidence” in the development of useful and relevant knowledge.

***Theme 2: Advancing IKT Methods***

**2.1 A Review Protocol on Research Partnerships: A Coordinated Multicenter Team Approach**

Hoekstra F, Mrklas KJ, Sibley KM, Nguyen T, Vis-Dunbar M, Neilson CJ, Crockett LK,

Gainforth HL, Graham ID

**Please go to the following link to view the published abstract**: <https://www.ncbi.nlm.nih.gov/pmc/articles/PMC6267881/>

**2.2 Conceptualizing integrated knowledge translation (IKT) initiation: a meta-narrative review**

Zych M, Berta W, Gagliardi A

**Background**

Integrated knowledge translation (IKT) refers to research-research user partnerships to co-generate and implement knowledge. IKT may be critical to IKT success, but has not been conceptualized. The purpose of this study was to conduct a meta-narrative review of IKT initiation concepts, processes, enablers, barriers and outcomes in health services research and in research from other disciplines and research traditions where IKT may be used.

**Methods**

Relevant research traditions were identified in several disciplines: social sciences (including psychology, education, and business), and healthcare (including medicine, nursing, public health, health services research). This information was used to develop eligibility criteria and a search strategy. Searches were conducted in MEDLINE, EMBASE, CINAHL, ABI Inform, ERIC, PsychInfo and the Cochrane Library from inception to June 9, 2017. Titles and abstracts were screened in triplicate; data were extracted in duplicate. Study characteristics, and conceptual and empirical findings associated each meta-narrative were tabulated, and summarized and compared.

**Results**

A total of 7,779 unique results were identified, 122 full-text items were examined, and 17 reviews published from 1998 to 2017 were eligible. Most reviews were conducted in the United States (n=7) or Canada (n=5); the most common type was a systematic review (n=6); and 10 and 7 emerged from the health care and social sciences literature, respectively. All reviews identified an IKT initiation phase referred to as “early” or “developmental”, or more vaguely as “fuzzy”, across six meta-narratives: Integrated knowledge translation, Action research, Stakeholder engagement, Knowledge transfer, Team initiation, and Shared mental models. The majority of IKT initiation processes, enablers, barriers and outcomes were common to multiple meta-narratives and summarized in an IKT Initiation Conceptual Framework. Proposed outcomes associated with IKT initiation reflected the relationship between researchers and research users (i.e. respect and trust, clear expectations) and project-specific outputs (i.e. research questions, project agenda). No research empirically demonstrated an association between IKT initiation and these outcomes.

**Conclusions**

While IKT initiation was recognized, it remains vaguely conceptualized despite lengthy research traditions. Ongoing research of IKT initiation is needed to identify or generate relevant theory, and to establish outcomes and the determinants of those outcomes.

**2.3 Digital Storytelling: A Methodology for Engaging Vulnerable and Marginalized Populations in Integrative Knowledge Translation**

Wazni L, Gifford W

**Background**

Integrated knowledge translation (iKT) focuses on partnerships between researchers and knowledge users to co-produce relevant and mutually beneﬁcial research findings. Co-production requires collaborative approaches that engage knowledge users in all stages of the research processes, from defining problems to formulating theories and implementing solutions. Knowledge users include policymakers, practitioners, patients (or their advocates), and members of the public and iKT requires participatory methodologies that promote collaborative decision-making and power sharing. However, these approaches are particularly challenging with vulnerable and marginalized groups who are typically perceived to have less power and voice in decision-making. Innovative approaches are required to engage marginalized groups in iKT research.

**Discussion**

In this paper we discuss digital storytelling, an innovative approach to bring the voices of vulnerable and marginalized populations. We present how visual methods of digital storytelling can provide an opportunity for marginalized people to describe, reflect, and share their health and health care experiences. We will also illustrate how the use of an IKT approach with digital storytelling can further enhance giving voice by ensuring people are involved in defining research problems, understanding their contexts, and developing impactful and meaningful solutions.

**Conclusion**

A powerful and creative form of communication and expression, digital storytelling can uncover and amplify marginalized peoples' voices for knowledge creation and translation. By using the power of participant-produced narratives and visual imagery, digital storytelling allows marginalized groups to represent themselves and integrate their experiences, knowledge and needs and the use of an integrated knowledge translation approach ensures the participation of these groups is inclusive, meaningful and directing the research focus.

**2.4 Community-based participatory research and integrated knowledge translation: advancing the co-creation of knowledge**

Jull J, Giles A, Graham I

**Background**

Better use of research evidence (one form of “knowledge”) in health systems requires partnerships between researchers and those who contend with the real-world needs and constraints of health systems. Community-based participatory research (CBPR) and integrated knowledge translation (IKT) are research approaches that emphasize the importance of creating partnerships between researchers and the people for whom the research is ultimately meant to be of use (“knowledge users”). There exist poor understandings of the ways in which these approaches converge and diverge. Better understanding of the similarities and differences between CBPR and IKT will enable researchers to use these approaches appropriately and to leverage best practices and knowledge from each. The co-creation of knowledge conveys promise of significant social impacts, and further understandings of how to engage and involve knowledge users in research are needed.

**Main text**

We examine the histories and traditions of CBPR and IKT, as well as their points of convergence and divergence. We critically evaluate the ways in which both have the potential to contribute to the development and integration of knowledge in health systems. As distinct research traditions, the underlying drivers and rationale for CBPR and IKT have similarities and differences across the areas of motivation, social location, and ethics; nevertheless, the practices of CBPR and IKT converge upon a common aim: the co-creation of knowledge that is the result of knowledge user and researcher expertise. We argue that while CBPR and IKT both have the potential to contribute evidence to implementation science and practices for collaborative research, clarity for the purpose of the research—social change or application—is a critical feature in the selection of an appropriate collaborative approach to build knowledge.

**Conclusion**

CBPR and IKT bring distinct strengths to a common aim: to foster democratic processes in the co-creation of knowledge. As research approaches, they create opportunities to challenge assumptions about for whom, how, and what is defined as knowledge, and to develop and integrate research findings into health systems. When used appropriately, CBPR and IKT both have the potential to contribute to and advance implementation science about the conduct of collaborative health systems research.

***Theme 3: Evaluation and Impact***

**3.1 Embracing complexity and uncertainty to create impact: exploring the emergence, processes and transformative potential of co-produced research**

Beckett K, Farr M, Kothari A, Wye L, le May A

The potential use, influence and impact of health research is seldom fully realised. This stubborn problem has caused burgeoning global interest in research aiming to address the implementation ‘gap’ and factors inhibiting the uptake of scientific evidence. Scholars and practitioners have questioned the nature of evidence used and required for healthcare, highlighting the complex ways that knowledge is formed, shared and modified in practice and policy. This has led to rapid expansion, expertise and innovation in the field of Knowledge Mobilisation (KM) and funding for experimentation into the effectiveness of different KM models. One approach gaining prominence involves stakeholders (e.g. researchers, practitioners, service users, policy-makers, managers and carers) in the co-production, and application, of knowledge for practice, policy and research (frequently termed Integrated Knowledge Translation in Canada). Its popularity stems largely from its potential to address dilemmas inherent in the implementation of knowledge generated using more reductionist methods. However, despite increasing recognition, demands for co-produced research to illustrate its worth are becoming pressing while the means to do so remain challenging. This is due not only to the diversity of approaches to co-production and their application, but also to the ways through which different stakeholders conceptualise, measure, reward and use research. While research co-production can lead to demonstrable benefits such as policy or practice change, it may also have more diffuse and subtle impact on relationships, knowledge sharing, and in engendering culture shifts and research capacity building. These relatively intangible outcomes are harder to measure and require new emphases and tools. This opinion paper uses six Canadian and UK case studies to explore the principles and practice of co-production and illustrate how it can influence interactions between research, policy and practice and benefit diverse stakeholders. In doing so, we identify a continuum of co-production processes. We propose and illustrate the use of a new ‘social model of impact’ and framework to capture multi-layered and potentially transformative impacts of co-produced research. We make recommendations for future directions in research co-production and impact measurement.

**3.2 Advancing the Evaluation of Integrated KT**

Kreindler SA

**Background**

Integrated knowledge translation (IKT) flows from the premise that knowledge co-produced with decision-makers is more likely to inform subsequent decisions. However, evaluations of manager/policymaker-focused IKT often focus on intermediate outcomes, stopping short of assessing whether research findings have contributed to identifiable organizational action. Such hesitancy may reflect the difficulty of tracing the causes of this distal, multifactorial outcome. This paper elucidates how an approach based on realistic evaluation could advance the field.

**Main Text**

Realistic Evaluation views outcomes as a joint product of intervention mechanisms and context. Through identification of context–mechanism–outcome configurations, it enables the systematic testing and refinement of "mid-range theory" applicable to diverse interventions that share a similar underlying logic of action. The "context-sensitive causal chain" diagram, a tool adapted from the broader theory-based evaluation literature, offers a useful means of visualizing the posited chain from activities to outcomes via mechanisms, and the context factors that facilitate or disrupt each linkage (e.g., activity–mechanism, mechanism–outcome). Drawing on relevant literature, this paper proposes a context-sensitive causal chain by which IKT may generate instrumental use of research findings (i.e., direct use to make a concrete decision) and identifies an existing tool to assess this outcome, then adapts the chain to describe a more subtle, indirect pathway of influence. Key mechanisms include capacity- and relationship-building among researchers and decision-makers, changes in the (perceived) credibility and usability of findings, changes in decision-makers' beliefs and attitudes, and incorporation of new knowledge in an actual decision. Project-specific context factors may impinge upon each linkage; equally important is the organization's absorptive capacity: its overall ability to acquire, assimilate, and apply knowledge. Given a sufficiently poor decision-making environment, even well-implemented IKT that triggers important mechanisms may fall short of its desired outcomes. Further research may identify additional mechanisms and context factors.

**Conclusion**

By investigating "what it is about an intervention that works, for whom, under what conditions," Realistic evaluation addresses questions of causality head-on without sacrificing complexity. A realist approach could contribute greatly to our ability to assess – and ultimately, to increase – the value of IKT.

**3.3 Exploring the Synergies between Focused Ethnography and Integrated Knowledge Translation**

Baumbusch J, Wu S, Lauck SB, Banner D, O’Shea T, Achtem L

**Background**

Issues with the uptake of research findings in applied health services research remain problematic. Part of this disconnect is attributed to the exclusion of knowledge users at the outset of a study, which often results in the generation of knowledge that is not usable at the point-of-care. Integrated knowledge translation (iKT) blended with qualitative methodologies has the potential to address this issue by working alongside knowledge users throughout the research process. To date, there is a paucity of literature about how iKT can be integrated with a qualitative methodology, and we begin to address this gap in this paper. The purpose of this paper is to describe our experience of conducting a focused ethnography with a collaborative iKT approach, including the synergies and potential sources of discord between iKT and focused ethnography.

**Methods**

We describe the specific characteristics and synergies that exist when engaging in iKT in a focused ethnography, using a research exemplar about the experiences of frail, older adults undergoing a transcatheter aortic valve implantation.

**Results**

Employing iKT in concert with focused ethnography resulted in (1) an increased focus on the culture and values of the context under study; (2) a higher level of engagement among researchers, participants, and knowledge users; (3) a commitment to partnership between researchers and knowledge users as part of a larger program of research, resulting in a (4) greater emphasis on the importance of reciprocity and trustworthiness in the research process.

**Conclusions**

Engaging in iKT from the outset of a study ensures that research findings are relevant for application at the point-of-care. The integration of iKT with focused ethnography allows for real-time uptake of meaningful, emerging findings, the strengthening of collaborative research teams, and opportunities for sustained programs of research and relationships in the field of health services research. Further exploration of the integration of collaborative approaches to iKT in qualitative methodologies is recommended.

**3.4 Variable participation of knowledge users in cancer health services research**

O’Brien MA, Carson A, Barbera L, Brouwers MC, Earle CC, Graham ID, Mittmann N, Grunfeld E.

**Background**

Integrated knowledge translation (IKT) is a research approach in which knowledge users (KUs) co-produce research. The rationale for IKT is that it leads to research that is more relevant and useful to KUs, thereby accelerating uptake of findings. The aim of the current study was to evaluate IKT activities within a cancer health services research network in Ontario, Canada.

**Methods**

An embedded multiple case study design was used. The cases were 5 individual studies within an overarching cancer health services research network. These studies focused on one of the following topics: case costing of cancer treatment, lung cancer surgery policy analysis, patient and provider-reported outcomes, colorectal cancer screening, and a team approach to women’s survivorship. We conducted document reviews and held semi-structured interviews with researchers, KUs, and other stakeholders within a cancer system organization. The analysis examined patterns across and within cases.

**Results**

Researchers and their respective knowledge users from 4 of the 5 cases agreed to participate. Eighteen individuals from 4 cases were interviewed. In 3 of 4 cases, there were mismatched expectations between researchers and KUs regarding KU role; participants recommended that expectations be made explicit from the beginning of the collaboration. KUs perceived that frequent KU turnover may have affected both KU engagement and the uptake of study results within the organization. Researchers and KUs found that sharing research results was challenging because the organization lacked a framework for knowledge translation. Uptake of research findings appeared to be related to the researcher having an embedded role in the cancer system organization and/or close alignment of the study with organizational priorities. Document reviews found evidence of planned IKT strategies in 3 of 4 cases; however, actual KU role/engagement on research teams was variable.

**Conclusions**

Barriers to KU co-production of cancer health services research include mismatched expectations of KU role and frequent KU turnover. When researchers are embedded in the cancer system organization and the project aligns with priorities, it appears more likely that results will be considered in programming. Research teams that take an IKT approach should consider specific strategies to address barriers to KU engagement.

**3.5 Translating research into action: an international study of the role of research funders**

Mclean R, Graham I, Tetroe JM, Volmink J

**Background**

It is widely accepted that research can lead to improved health outcomes. However, translating research into meaningful impacts in peoples’ lives requires actions that stretch well beyond those traditionally associated with knowledge creation. The research reported in this manuscript provides an international review of health research funders’ efforts to encourage this process of research uptake, application and scaling, often referred to as knowledge translation.

**Methods**

We conducted web-site review, document review and key informant interviews to investigate knowledge translation at 26 research funding agencies. The sample comprises the regions of Australia, Europe and North America, and a diverse range of funder types, including biomedical, clinical, multi-health domain, philanthropic, public and private organisations. The data builds on a 2008 study by the authors with the same international sample, which permitted longitudinal trend analysis.

**Results**

Knowledge translation is an objective of growing significance for funders across each region studied. However, there is no clear international consensus or standard on how funders might support knowledge translation. We found that approaches and mechanisms vary across region and funder type. Strategically tailored funding opportunities (grants) are the most prevalent modality of support. The most common funder-driven strategy for knowledge translation within these grants is the linking of researchers to research users. Funders could not to provide empirical evidence to support the majority of the knowledge translation activities they encourage or undertake.

**Conclusions**

Knowledge translation at a research funder relies on context. Accordingly, we suggest that the diversity of approaches uncovered in our research is fitting. We argue that evaluation of funding agency efforts to promote and/or support knowledge translation should be prioritised and actioned. It is paradoxical that funders’ efforts to get evidence into practice are not themselves evidence based.

***Theme 4: Patient Engagement***

**4.1 Patient Engagement in Integrated Knowledge Translation Research**

Banner D, Carroll S, Fyfe T, Gagliardi A, Kandola, D, Rolfe D, Wong C, Graham ID

**Background**

Over recent decades, healthcare organizations across the world are being increasingly challenged to develop and implement services that are evidence-based and bring about improvement in patient and health service outcomes. Despite an increasing emphasis upon evidence-based practice, large variations in practice remain and gaps pervade in the creation and application of knowledge that improves outcomes. The meaningful engagement of patients, in addition to the inclusion of patient-reported outcomes and priorities, has been hailed as one mechanism to improve the relevance, impact and efficiency of research. This cultural shift is reflected in key research strategies and programs, including the Canadian Institutes of Health Research (CIHR) Citizen Engagement Framework, Health Research Roadmap II and Strategy for Patient Orientated Research (SPOR). Knowledge Translation (KT), coined by the CIHR, “is a dynamic and iterative process that includes synthesis, dissemination, exchange and ethically sound application of knowledge to improve the health of Canadians, provide more effective health services and products and strengthen the health care system” (CIHR, 2012). While the notion of KT is widely adopted, there are multiple terms that may be used interchangeably, such as knowledge exchange and transfer, and similarly diverse applications of these principles in health research. Integrated KT (IKT) is a collaborative model of research that engages potential knowledge users, including decision makers, healthcare providers, policy makers and the public, as partners within the research. Separate from end-of-grant KT, integrated KT is a collaborative model and process through which knowledge users and researcher work together across the research process to identify key priorities, develop responsive research questions, interpret findings and apply outcomes to practice. Collectively, both IKT and patient engagement processes provide a vehicle to address health disparities and improve the delivery of effective and responsive healthcare services. However, while one could argue that these are inextricably connected through their engagement focus, it is unclear how IKT and patient engagement processes are linked conceptually and in practice.

**Goals/Methods**

The purpose of this debate paper is to explore how patients are engaged in IKT research and explore how IKT approaches can foster meaningful patient engagement. We will explore how IKT and patient orientated research are conceptually similar and distinct and will draw upon the existing literature to highlight key examples. To achieve this, we will replicate and extend a recent scoping review exploring IKT in healthcare (Gagliardi *et al.* 2016) with the specific lens of explore how and where patients were engaged. In concert, we will consult with key experts in IKT and patient engagement research to seek consensus on the emerging conceptual data.

**Proposed Outcomes**

By undertaking this iterative and exploratory process, we aim to provide greater conceptual clarity and density around patient engagement within IKT research and to make explicit how the principles, practices and outcomes of patient engagement and IKT overlap across the research process. This provides the opportunity to take a critical look at two dominant approaches and models and to identify potential gaps or areas for further development and research. By understanding this further, it is possible that collaborative teams may be better able to develop and undertake research that fosters the meaningful engagement of patients and further incorporates outcomes of interest to patients and communities. In addition, we hope to generate examples of the actual or potential impact, process, and outcomes of patient engagement within IKT research.

**4.2 Patient Engagement and Integrated Knowledge Translation Research: What can be learned from qualitative health research methods?**

Rolfe D, Ramsden V, Banner-Lukaris D, Graham I

**Background**

The Canadian Institutes for Health Research (CIHR) Strategy for Patient Oriented Research (SPOR) has as its vision, “Support for People and Patient-Oriented Research and Trials (SUPPORT) Units will ‘conduct and support implementation science and integrated knowledge translation approaches” via the collaborative leadership of patients, researchers, policy makers, funders and health care professionals. Patient engagement (or the co-creation of research with patients) is an integral component of SPOR and its SUPPORT Units, and is positioned by CIHR as a promising and innovative approach to research that has been successfully adopted in other countries. What is missing from the recent proliferation of resources and publications detailing the practical and experiential aspects of patient engagement, however, is a recognition of the many existing research theories and methodologies that have an established history of public and patient involvement.

**Discussion**

The purpose of this paper is to explore how existing health research methodologies can inform current approaches to patient engagement, and support meaningful engagement with patients in integrated knowledge translation research. Specifically, this paper addresses issues of: rigour (how can patient engagement in research be done well?); representation (are the right patients being engaged?); and, reflexivity (is engagement being done in ways that are meaningful, ethical and equitable?). Various qualitative research methods (e.g., purposive sampling, theoretical saturation, member checking) are presented to increase the rigour found within patient engagement. Approaches to engage more diverse patient perspectives are presented to improve representation beyond the common practice of selectively engaging only one or two patients. Reflexivity, the practice of identifying and articulating how research processes and outcomes are co-constructed by the respective personal and professional experiences of researchers and patients, is presented to support the development of authentic, sustainable, equitable and meaningful engagement of patients in research.

**Conclusions**

Integrated knowledge translation researchers will increasingly need to engage patients as stakeholders in order to satisfy the overlapping mandate in health policy, care and research to engage patients as partners in decision-making. This paper suggests several suggestions to ground patient engagement approaches and practices in established research methodologies, theories and approaches.

**4.3 Engaging Frail and Seriously Ill Patients in Integrated Knowledge Translation: A Systematized Review**

Ludwig C, Stacey D, Graham I, Backman c, Gifford W

Patients are increasingly recognized as essential partners in research, not only providing unique insight into their lived experience of illness but determining what and how research should be done. However, it appears that patients who are frail and seriously ill are not involved as research partners to the same extent as patients who are living with more stable illness. This review explored how those with serious illness and frailty have been involved as research partners; specifically, the practical and ethical issues associated with participation throughout the course of a research project, and the benefits of engaging them.

Given the fluctuations in health for frail and seriously ill patients, researchers need to be flexible and creative in their approach to patient engagement. Potential risks and challenges need to be outweighed with benefits to the patient, the researcher, and the research itself. Patients benefit from a renewed sense of purpose in the face of debilitating or life-limiting disease, gaining emotional support and acquiring new skills. Researchers describe finding new ways of working and an improvement in their inter-personal skills. Research produced with patients is more relevant because it addresses their needs and concerns. Further research is required in order to build the evidence base about what it means to engage frail and seriously ill patients in research because their knowledge is essential in developing research that is more applicable to their needs.
